# The Chalk Streams of Southern England and Northern France Harbour Substantial Unique Components of the Overall Genetic Diversity of Atlantic Salmon ( *Salmo salar* L.)

**DOI:** 10.1111/eva.70237

**Published:** 2026-04-17

**Authors:** R. Andrew King, Guillaume Evanno, Jamie R. Stevens

**Affiliations:** ^1^ Department of Biosciences, Faculty of Health and Life Sciences, Hatherly Laboratories University of Exeter Exeter UK; ^2^ DECOD (Ecosystem Dynamics and Sustainability), INRAE, Institut Agro, IFREMER Rennes France; ^3^ Penrose Ecological Ltd Exeter UK

**Keywords:** Atlantic salmon, conservation units, genetic structure, management priorities, SNP

## Abstract

Populations of Atlantic salmon continue to suffer marked declines in abundance due to stressors acting in both their freshwater and marine habitats. It is therefore an imperative to identify populations in need of increased conservation intervention, with the aim of preserving as much as possible the genetic diversity present within the species. Previous microsatellite‐based analyses have shown the chalk rivers of southern England and northern France to hold genetically distinct populations of salmon. However, these salmon populations have never been investigated in the same study. Using a suite of 93 single nucleotide polymorphism loci and samples from 42 British Isles and French rivers, we demonstrate the French and English chalk salmon to be closely related and confirm their distinction from salmon inhabiting non‐chalk rivers. The identification of a small number of significant *F*_ST_ outliers and loci associated with environmental variables suggests that this distinction is driven by local adaptation. We propose that the chalk and non‐chalk salmon be designated as two distinct Evolutionarily Significant Units that each contain multiple Management Units. The chalk river salmon, especially those from southern England, are identified as making a significant contribution to the overall diversity of the species within the English Channel region. Accordingly, we suggest that the salmon populations of the chalk streams could be considered as meeting the criteria for recognition as a distinct subspecies of salmon, *Salmo salar calcariensis*. Taken together, our findings highlight the urgent need for a better understanding of the intraspecific diversity of the species and the importance of such information in enhancing the conservation and protection of Atlantic salmon populations inhabiting the chalk rivers of southern England and northern France.

## 1 Introduction

Freshwater biodiversity is in a state of global crisis (Dudgeon 2019). Increasingly it is becoming clear that anthropogenic activities are having adverse effects on a wide range of ecological and evolutionary processes, leading to drastic and accelerating declines in size of wildlife populations (WWF 2024). The consequences of these activities are being felt in all terrestrial and aquatic habitats but especially so in freshwater habitats (Dudgeon 2019; WWF 2024) through the multiple, co‐occurring stressors of habitat degradation and loss, both point and diffuse source pollution, exploitation of water resources, that is, over abstraction, invasive species and climate change (Dudgeon 2019; Haase et al. 2025). The negative effects of stressors on species and ecosystems can lead to losses of genetic diversity, changes in species ranges and species extinctions (Comte et al. 2013; Exposito‐Alonso et al. 2022; Shaw et al. 2025; Urban 2024). Globally, populations of migratory fish have shown particularly striking declines in abundance with reductions in excess of 80% over the last 50 years, with the most marked declines occurring since the mid‐1990s (Deinet et al. 2024). Within Europe, species abundances have declined by 75% since the mid‐1990s. Atlantic salmon ( *Salmo salar* ) is an iconic anadromous fish species. After spending one to several years in freshwater, salmon smoltify and migrate to sea to feed and grow, usually for one to three years, before returning to their natal river to spawn (Crisp 2000; Klemetsen et al. 2003). The species has suffered marked range‐wide declines due to the combined effects of stressors in both its freshwater natal and spawning habitat and its marine feeding habitats (Dadswell et al. 2022; Gillson et al. 2022; Nunn et al. 2023). Recently, the IUCN have updated the conservation status of Atlantic salmon, downgrading the species globally to near threatened. Assessments were also published for different genetic groups within the species, with the salmon populations of Britain classified as endangered, the English chalk stream populations as vulnerable, the French Allier sub‐population as endangered and the France/Spain grouping as of least concern (Darwell 2023a, 2023b, 2023c, 2023d; Evanno et al. 2023).

In a natural world increasingly affected by the detrimental actions of humans, it is important to identify populations in need of increased conservation management and to safeguard as many populations as possible to meet recent United Nations targets of protecting at least 90% of diversity present within a species (Exposito‐Alonso et al. 2022). Genetic and genomic data have been used extensively to help inform conservation management decision‐making processes (Forrester and Lama 2023). For some species, the conservation of genetically distinct populations, which can represent unique evolutionary lineages, has been prioritised (Chhina et al. 2024; von Takach et al. 2024).

Chalk streams are a globally rare habitat found predominantly in southern and eastern England but also in northern France and Denmark (CABA 2021; WWF‐UK 2015). Such watercourses are dominated by groundwater flowing from Cretaceous‐age chalk aquifers (Berrie 1992; Sear et al. 1999). The chalk has a strong influence on the flow regime, temperature and chemical properties of the river water, resulting in chalk streams supporting characteristically distinctive plant and animal assemblages (Berrie 1992; Sear et al. 1999).

Several studies have shown that the salmon populations inhabiting the chalk streams of the Hampshire Basin in southern England are one of the most genetically unique groups of rivers in the northeast Atlantic distribution of Atlantic salmon (Finnegan et al. 2013; Gilbey et al. 2018; Griffiths et al. 2010; Ikediashi et al. 2018). Likewise, in northern France, the salmon populations from the rivers of Upper Normandy, which also flow over chalk substrate, are among the most distinct within the French distribution of the species (Perrier et al. 2011). These studies, based on analysis of microsatellite loci, have generally shown lower levels of genetic diversity in chalk rivers compared to neighbouring rivers flowing over non‐chalk substrates (Finnegan et al. 2013; Ikediashi et al. 2018; Perrier et al. 2011). However, the Hampshire Basin and Upper Normandy salmon populations have never been investigated in the same study. Moreover, being outside the main range of Atlantic salmon in western Europe (Scotland, Ireland and Norway), coverage of English Channel populations has been sparse in recent range‐wide single nucleotide polymorphism (SNP) based studies of the species (Bradbury et al. 2021; Jeffery et al. 2018; O'Sullivan et al. 2022).

Here, we explore patterns of genetic diversity in Atlantic salmon from 42 rivers from the southern British Isles and northern France using a recently developed panel of SNP markers (King and Stevens 2021), with an emphasis on populations in the chalk rivers of the Hampshire Basin and Upper Normandy. Our objectives were to (1) assess patterns of genetic diversity and structuring of salmon within these rivers, (2) determine the relationship between salmon inhabiting the chalk rivers of southern England and northern France, and populations in non‐chalk rivers, and (3) identify salmon populations in need of targeted conservation management.

## 2 Materials and Methods

### 2.1 Sample Collection

**FIGURE 1:**
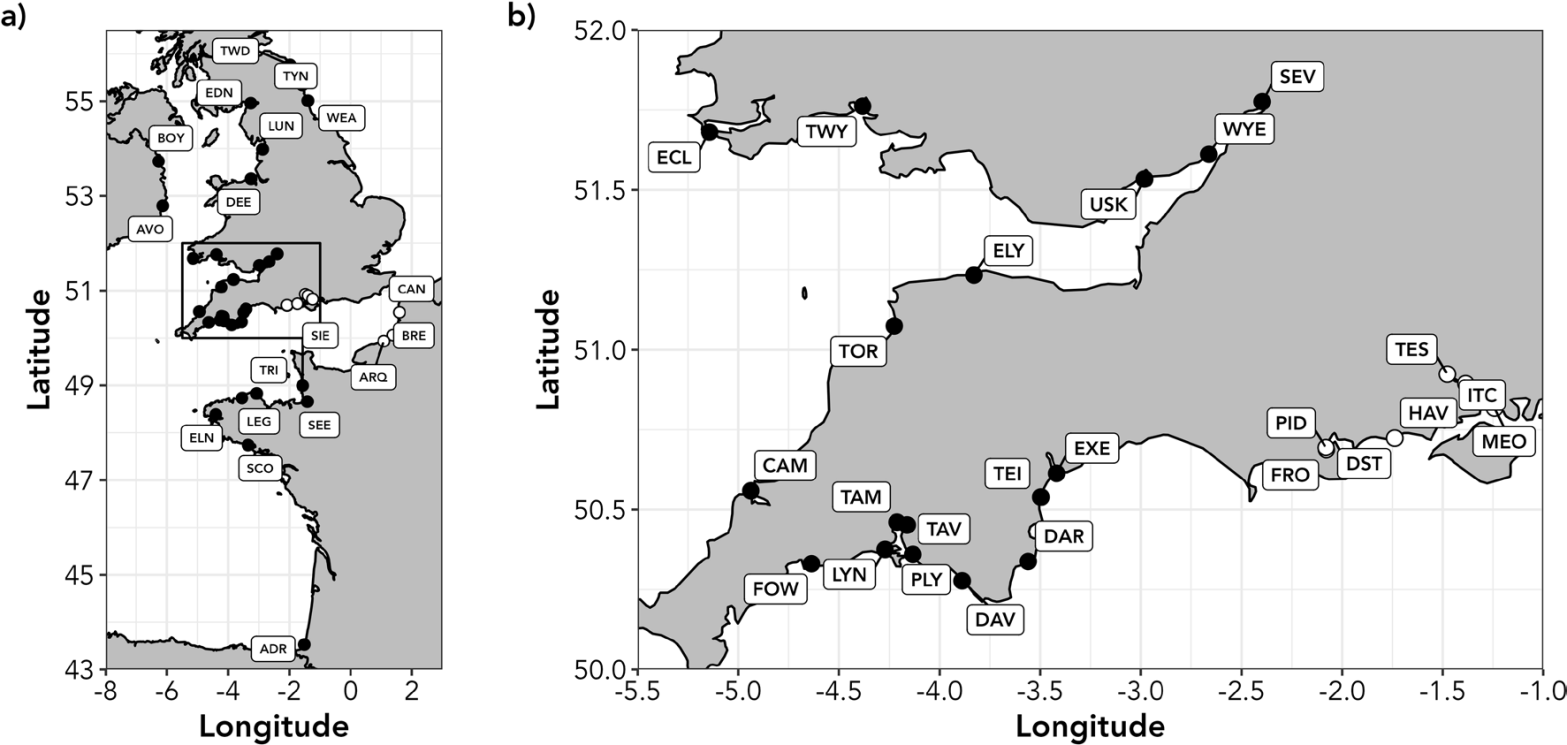
Map showing the sampled Atlantic salmon rivers in Britain, Ireland and France: (a) location of the 42 sampled rivers, (b) detail of rivers in southern England and Wales. For clarity, only the location of the river mouth is shown. White and black symbols represent chalk and non‐chalk rivers, respectively. River codes are as given in Table S1.

Individual Atlantic salmon were sampled from 42 English, Irish, and French rivers, the majority of which are at high risk of local extirpation (Figure 1, Table S1). The majority of fish were caught as fry or parr during routine management surveys by the Environment Agency, Natural Resources Wales, Inland Fisheries Ireland, and the Game and Wildlife Conservation Trust. Collections typically consisted of samples collected during electrofishing surveys from multiple sites across each river catchment. Fish were anaesthetised using 2‐phenoxyethanol or MS‐222 prior to removal of tissue samples (either fin clips or scales). Fin clips were transferred immediately into tubes containing absolute ethanol. Scale samples were also obtained from trap‐ or rod‐caught adults from the Tamar, Wye, and northern French rivers. Genomic DNA was extracted from fin clips following the method of Truett et al. (2000) and scales using Qiagen DNeasy Blood and Tissue kits, following the manufacturer's instructions.

### 2.2 SNP Genotyping

Each sample was screened for variation at 94 biallelic single nucleotide polymorphism (SNP) markers recently developed for Atlantic salmon (King and Stevens 2021). Full details on the development of the SNP assays are given in King and Stevens (2021). Details on the nature of each SNP (i.e., coding or non‐coding) and their location within the Atlantic salmon genome are given in Table S2. The majority of individuals were genotyped under the present project with a small number of genotypes taken from King and Stevens (2021). SNP genotyping was undertaken on a Standard Biotools EP1 Genotyping System using 96.96 Dynamic Genotyping Arrays and scored using the Standard Biotools SNP Genotyping analysis software. Each genotyping run included two positive (individuals of known genotype) and two negative controls. Genotype plots were manually inspected to ensure quality of individual genotyping and clustering. Samples deemed to be outside the ‘normal’ range of the homozygous and heterozygous genotype clusters for an assay were scored as ‘No Call’. Genotyping error rate was assessed by repeated genotyping of 26 individuals on different genotyping runs and was calculated as the number of allele call mismatches/total number of allele calls.

### 2.3 Data Quality Control

Call rate per individual and per locus was assessed using the propTyped() function in the adegenet r package (Jombart 2008). Salmonid populations can often contain numbers of closely related individuals (Hansen et al. 1997), the presence of which can potentially lead to bias in population genetic parameter estimation (Goldberg and Waits 2010) and false inference of population structure (Rodríguez‐Ramilo and Wang 2012). For sibship reconstruction, we used COLONY v 2.0.5.9 (Jones and Wang 2010), which implements a maximum‐likelihood method to assign sibship to individuals using their multilocus genotype. Run conditions were: high precision, medium length run, assuming both male and female polygamy without inbreeding, and a conservative 0.5% error for both scoring error rate and allelic dropout rate. Fish were considered members of a full‐sib family if the probability of exclusion was > 0.9. We used the Waples and Anderson (2017) Yank‐2 method to trim full sibs from the data set—both members of families with two individuals were retained but if a family had three or more individuals, all but two random members were removed.

Genepop v 3.4 (Raymond and Rousset 1995) was used to test for linkage disequilibrium (LD) between all pairs of loci within each population. Significance was estimated using a Markov‐chain method (1000 de‐memorisations, 100 batches and 1000 iterations). Hochberg's (1988) correction was used to adjust significance levels for multiple comparisons (https://www.multipletesting.com, Menyhart et al. (2021)). Deviation from Hardy–Weinberg Equilibrium (HWE), based on Nei's (1987) heterozygosity‐based G_IS_ estimator, for each locus and population was tested using GenoDive v3.03 (Meirmans 2020). Significance was based on 999 permutations.

### 2.4 Genetic Diversity

Basic measures of genetic diversity (H_O_—observed heterozygosity and H_S_—unbiased expected heterozygosity) and Weir and Cockerham's (1984) estimator of *F*_ST_ were calculated using GenoDive v3.03 (Meirmans 2020). Significance of *F*_ST_ values was assessed using 999 bootstrap replicates. The percentage of polymorphic loci in each collection after subsampling to the smallest number of genotyped fish was calculated using the R scripts of Anderson et al. (2025).

### 2.5 Selection Tests

#### 2.5.1 *F*_ST_ ‐Based Outlier Tests

Two *F*_ST_‐based tests were performed to identify loci under selection. Bayescan 2.01 (Foll and Gaggiotti 2008) implements the Bayesian approach of Beaumont and Balding (2004). The program was run under default settings (20 pilot runs of 5000 iterations each followed by an additional burn‐in of 50,000 iterations and then 50,000 samplings with a thinning interval of 10). We complemented the Bayescan results with simulations using the fdist model as implemented in Arlequin 3.5 (Excoffier and Lischer 2010) using default parameters (10,000 simulations with a null model with 10 groups, each containing 100 demes). Both Bayescan and fdist analyses were run on three separate datasets—full (42 rivers, 93 SNPs), non‐chalk (32 rivers, 89 SNPs) and chalk (10 rivers, 88 SNPs) datasets. Additionally, hierarchical genetic structuring can lead to an excess of false positives if not accounted for in outlier tests (Excoffier et al. 2009). We therefore repeated the fdist analysis on the full dataset using the hierarchical *F*_ST_ model, based on two data partitions: chalk v non‐chalk, and UK chalk v FR chalk v UK non‐chalk v FR non‐chalk. Loci were considered under selection if they were outside of the 99% confidence level of the simulated neutral distribution.

#### 2.5.2 Genotype‐Environment Associations

To investigate associations between genetic data and environmental variables we performed two analyses. Firstly, a multivariate Redundancy Analysis (RDA), as implemented in the *vegan* v2.5‐2 R package (Oksanen et al. 2019), was run. Genetic data was the minor allele frequency (MAF) at each SNP locus. Environmental data for each river, in the form of 19 bioclim variables at 1 km spatial resolution, were extracted from WorldClim databases using the *raster* v3.0‐7 R package (Fick and Hijmans 2017; Hijmans 2019). For rivers where fish were sampled from multiple sites, we used the data for the furthest downstream site. For the northern French rivers, where samples were collected from rod‐caught adults from across each catchment, we chose a point ~10 km upstream of the river mouth as the environmental site. The environmental data were standardised using the base R scale() function. To account for significant correlations between the bioclim variables we initially ran the RDA with all 19 variables and progressively removed the variable with the highest variance inflation factor (vif) until all remaining vif values were less than 10. Analysis of Variance (ANOVA), based on 999 permutations, was used to test the significance of the full RDA model, each RDA axis and each bioclim variable. Candidate SNP loci were identified in the tail of the allele loading distribution for each axis, using a 3‐standard deviation (SD) from the mean loading cut‐off (corresponds to a two‐tailed *p*‐value = 0.0027—Forester et al. (2018)). A second RDA was run conditioned on geographical distance from the northern‐most river (the Tweed). Least‐cost marine distances between the mouth of each river and the Tweed were calculated using the marmap R package (Pante and Simon‐Bouhet 2013).

Secondly, we ran BayeScEnv (de Villemereuil and Gaggiotti 2015) which implements a Bayesian *F*_ST_‐based univariate method, incorporating environmental information, to test for genotype‐environment associations. We ran the analyses using default settings: 20 pilot runs of 5000 iterations each followed by an additional burn‐in of 50,000 iterations and then 50,000 samplings with a thinning interval of 10. To minimise false positive associations, the prior probability was set to 0.1. The environmental inputs were the standardised data for each bioclim variable. Tests for convergence and auto‐correlation were conducted in the r package CODA (Plummer et al. 2006). A SNP was accepted as being associated with an environmental variable if the q‐value was less than 0.05.

For SNP loci identified as outliers in *F*_ST_‐based tests and showing significant associations with environmental data, we identified the genome location of each locus. The original RADtag sequences (King and Stevens 2021) were aligned to the Atlantic salmon genome (Ssal_v3.1) using the BLAST option in SalmoBase (Samy et al. 2017). We noted linkage group, location and whether the SNP was present in a gene or non‐coding region.

### 2.6 Genetic Structure

To investigate genetic structuring, we performed three analyses. Firstly, using POPULATIONS v1.2.32 (Langella 1999), a neighbour‐joining dendrogram based on Cavalli‐Sforza and Edwards (1967) chord distance (D_CE_) was constructed and visualised using MEGA v6 (Tamura et al. 2013). Secondly, data were analysed using the Bayesian‐based Markov Chain Monte Carlo (MCMC) model‐based clustering method implemented in STRUCTURE v 2.3.4 (Pritchard et al. 2000), which jointly defines *K*, the number of possible partitions of the data set and the proportion of an individual's genome (q) derived from each of the *K* partitions. STRUCTURE was run for 250,000 iterations following a burn‐in of 100,000 iterations with the number of inferred populations (*K*) ranging from one to ten. Ten independent runs at each *K* were performed using the admixture model with correlated allele frequencies and not using the river of origin information as a prior. The most likely number of clusters was determined using the Δ*K* method of Evanno et al. (2005). To identify finer‐levels of structure, hierarchical analyses were performed based on the Δ*K* results for the full data set. Consensus data were visualised using POPHELPER v1.0.6 (Francis 2017). Finally, Discriminant Analysis of Principal Components (DAPC; Jombart et al. (2010)) analyses were undertaken using the *adegenet* (Jombart 2008) package for R (R Core Team, 2018). The optim.a.score() function was used to select the optimum number of principal components to be retained in the analysis. As with the STRUCTURE analysis, hierarchical analyses were performed based on the results for the full dataset. For hierarchical analyses, loci found to be near monomorphic in either chalk or non‐chalk rivers were removed (five and four loci, respectively) from the datasets (Table S3).

### 2.7 Identification of Conservation Units and Conservation Priority

A key aim of conservation genetics is to highlight populations within a species that may require special conservation measures. We used multiple approaches to identify groups of rivers in need of urgent conservation intervention. Firstly, using the three‐step approach of Funk et al. (2012), we delineated Conservation Units (CUs) for the sampled rivers. Step 1 uses the information from all loci to identify Evolutionarily Significant Units (ESUs). Then, using data from neutral loci, demographically independent Management Units (MUs) were defined. Finally, adaptively differentiated units (AUs) were recognised using outlier loci. To determine the population groupings, we constructed population‐based neighbour‐joining dendrograms, as described above, based on 93 loci for the full, 81 loci for the neutral and 12 loci for the outlier datasets, respectively. The outlier dataset contained genotypes for the 12 loci found to be under selection in the Bayescan, fdist and fdist hierarchical analyses.

Finally, we estimated the expected contribution of each river to two diversity measures—gene and allelic diversity using the approaches of Caballero and Rodríguez‐Ramilo (2010) and Petit et al. (1998) as implemented in METAPOP2 (López‐Cortegano et al. 2019). The contribution of a river to within subpopulation (*H*_*S*_, *A*_*S*_), between subpopulation (*D*_*G*_, *D*_*A*_) and total (*H*_*T*_, *A*_*T*_) gene and allelic diversity, respectively, was estimated by serial removal of each river from the dataset and calculating the change in each diversity metric. Rivers can have either a negative or positive contribution to each diversity measure, with a positive contribution signifying a loss of diversity after removal of a river and an increase in diversity indicating a negative contribution. We also calculated each diversity measure for the four main population groupings identified in our genetic structure analyses.

## 3 Results

### 3.1 Data Quality

A total of 1682 individuals were genotyped at the 94 SNP loci (Table S1). Genotyping failed at six or more loci for 42 individuals, which were removed from all analyses. Overall, genotyping success per locus was high (average 99.68%, range 91.63%–100%). Comparison of genotypes from repeated samples gave an error rate of 0.0014% (14 mismatches from 9776 allele calls). All loci were polymorphic in at least ten of the sampled rivers, with levels of polymorphism ranging from 81.7% to 93.4% (Table S1). COLONY analysis found a total of 100 full sib families (range 0–9 families per sample and 2–19 members per family). A total of 75 full sibs were trimmed, leaving a final data set of 1565 salmon on which all subsequent analyses were performed.

Tests found a total of 34 significant cases of linkage between pairs of markers, with the majority in chalk rivers. Twelve and six of these cases involved marker pairs Ssa_25077–Ssa_1354 and Ssa_25077–Ssa_41749, respectively. Consequently, the data for Ssa_25077 were removed, leaving a final data set of 93 SNP markers.

There were 142 significant deviations from HWE (out of a total of 3906 river/locus combinations). None of these significant results were consistent across loci or rivers. Seven rivers showed significant deviations from HWE (Table S1), with three rivers (Meon, Sienne and Scorff) showing a significant heterozygote deficiency and four (Wear, Tamar, Dart and Stour) showing a significant excess of heterozygotes. Overall, all rivers and remaining loci were retained for further analyses.

### 3.2 Genetic Diversity

Basic measures indicated marked differences in genetic diversity between chalk and non‐chalk salmon rivers (Table S1). Both observed and expected heterozygosity were generally higher in the western non‐chalk and French chalk rivers and lowest in the Hampshire Basin chalk stream populations (Table S1). The lowest H_S_ values were found in salmon in the East Lyn and Meon (Table S1) – two rivers with very small salmon populations. After accounting for sample size, the Dorset Stour had the lowest percentage of polymorphic loci while the highest values were found in the Upper Normandy rivers (Table S1).

### 3.3 Loci Under Selection

#### 3.3.1 *F*_ST_ ‐Based Outlier Tests

**FIGURE 2:**
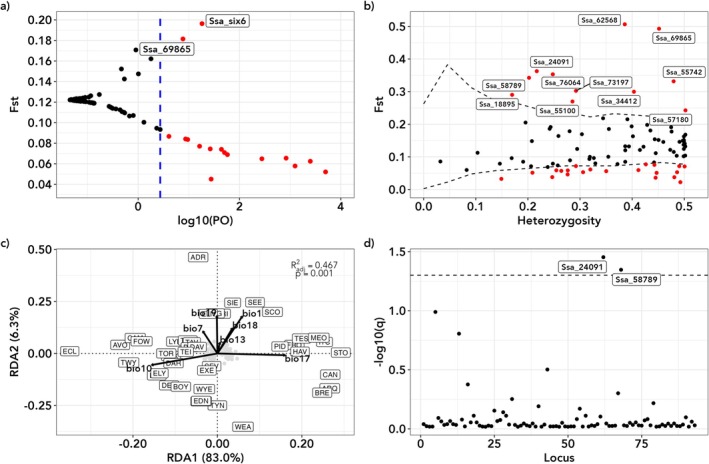
Results of selection and environmental association tests (a) Bayescan. The dashed blue line represents the log10 PO value above which loci are considered significant ouliers. (b) fdist results. Dashed lines are the upper and lower 99% confidence level of the simulated neutral distribution. Loci under either divergent or balancing selection are shown in red with loci under divergent selection labelled with locus name. (c) Plot of unconditioned redundancy analysis axes 1 v 2. Black vectors represent loadings for the retained bioclim variables. River codes are as given in Table S1. (d) Plot of ‐log10 q‐value from the BayeSceEnv outlier test for bio16—precipitation of the wettest quarter. The dotted line represents a q‐value of 0.05 (−log10 = 1.30). Loci above this line are significantly associated with the environmental variable in question.

Selection tests found a small number of loci under divergent selection in the three data sets. For the full data set, Bayescan and fdist found two and 11 loci, respectively, under divergent selection, with only a single locus (Ssa_69865) common to both analyses (Figure 2). Eleven of the outlier loci showed clear frequency differences between chalk and non‐chalk rivers (Figure S2), with the remaining locus (Ssa_six6) demonstrating high MAF in the Bristol Channel rivers (Usk, Wye & Severn). For the non‐chalk rivers, Bayescan and fdist found one and three loci, respectively, under divergent selection, with only a single locus (Ssa_six6) common to both analyses (Figure S1). For the chalk‐only data set outlier loci were only found in the fdist analysis with five loci identified as outliers (Figure S1). Considering multiple groups of rivers, the hierarchical fdist *F*_ST_ model found two SNPs, including Ssa_69865, under divergent selection in the analysis considering four groups of rivers (Figure S3). Both SNPs showed striking allele frequency differences between the chalk and non‐chalk river populations, being polymorphic in chalk rivers but near monomorphic in non‐chalk salmon populations (Figure S2).

The 12 *F*_ST_ outlier loci were found on ten linkage groups, with eight of the loci being located in introns of known genes (Table S2). Three loci were present in non‐coding regions and a single locus was found in a putative pseudogene.

#### 3.3.2 Genotype‐Environment Associations

For the final RDA model, seven bioclim variables with vif values under ten were retained; temperature variables bio1, bio7 and bio10 and precipitation variables bio13, bio17, bio18 and bio19. Both unconditioned and geographic distance conditioned analyses gave very similar results. In both cases the full model was highly significant (unconditioned—*F* = 5.20, *p* = 0.001, adjusted *r* ^2^ = 0.467; conditioned—*F* = 5.43, *p* = 0.001, adjusted *r* ^2^ = 0.409). Axis 1 separated the chalk rivers from non‐chalk rivers with this distinction being driven by mean temperature of the warmest quarter (bio10) and precipitation of the driest quarter (bio17). Axis 2 highlighted the Lower Normandy, Brittany and southern French rivers with this distinction being driven mainly by annual mean temperature (bio1) and precipitation of the coldest quarter (bio19) (Figure 2, Figure S4).

For the unconditioned analysis a single locus, Ssa_67740, was found to be associated with bio17 (Figure S4). The conditioned analysis also identified the Ssa_67740–bio17 association along with two additional loci—Ssa_30724 and Ssa_87179 associated with bio17 and bio10, respectively (Figure S4). Of the three SNPs associated with environmental variables, only one (Ssa_67740) was located in a known gene; Ssa_87179 was located in a predicted gene and Ssa_30724 was found in a non‐coding region (Table S2).

The BayeScEnv analyses identified two loci (Ssa_24091 and Ssa_58789) as being associated with bio16—precipitation of the wettest quarter (Figure 2, Figure S5). Ssa_24091 is located in an intron of the serine/threonine‐protein kinase 35, while Ssa_58789 is found in an intron of the insulin‐like growth factor 2 receptor. Both loci were also identified as outliers in the fdist analysis (Table S2).

### 3.4 Genetic Structuring

Population pairwise *F*_ST_ (Table S4, Figure S6) values ranged from −0.003 (Sienne v Sée) to 0.326 (Eden v Avon). All but five pairwise values where highly significant (Table S4). Within the non‐chalk river populations, average pairwise *F*_ST_ was 0.044 (range −0.003–0.141) despite very large geographic distances between some rivers (i.e., Tweed—Adour, least‐cost geographic distance = 1761 km; *F*_ST_ = 0.08). Within the chalk group average pairwise *F*_ST_ was 0.033 (range 0.002–0.067). Between non‐chalk and chalk rivers, pairwise differentiation values were all greater than 0.145 (average = 0.254, maximum = 0.326).

**FIGURE 3:**
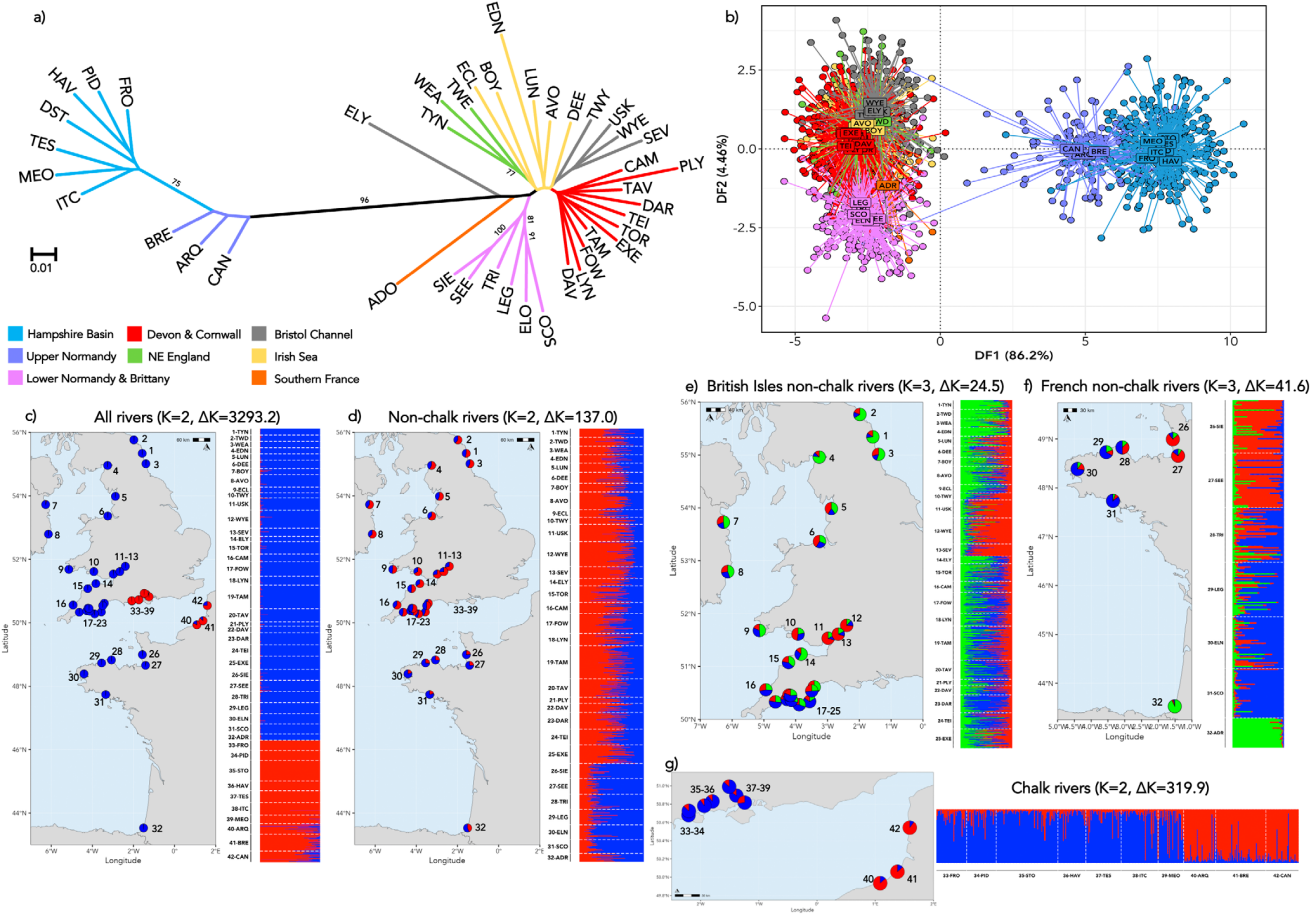
Results of the genetic structuring analyses for Atlantic salmon sampled from 42 British, Irish and French rivers. (a) unrooted neighbour‐joining dendrogram, based on Cavalli‐Sforza and Edwards' chord distance; branches are colour coded by geographical location. (b) Discriminant Analysis of Principal Components analysis for 1565 Atlantic salmon showing discriminant function 1 v 2; each dot represents a sampled individual fish and are coloured as in (a). (c–g) Results for hierarchical STRUCTURE analyses; results of each analysis are shown as bar plots with horizontal columns representing the assignment probabilities of individuals to each of the K inferred clusters. Maps show the location of each sampled river mouth with pie charts giving the river‐level assignment to each genetic cluster. Plots of ΔK values for STRUCTURE analyses are given in Figure S6. River codes are as given in Table S1.

The results of the neighbour‐joining, STRUCTURE and DAPC analyses were in broad agreement. All three analyses confirmed the strong split between non‐chalk and chalk river populations seen with pairwise *F*_ST_ values (Figure 3). For the STRUCTURE analysis, the Δ*K* method identified *K* = 2 as the most likely partition of the full data set (Δ*K* = 3293.2—Figure S7), differentiating the salmon from the chalk rivers of the Hampshire Basin and Upper Normandy rivers from all others. There was significant admixture between the non‐chalk and chalk groups within the Arques, Bresle and Canche rivers, with an average ancestry contribution of 0.178 from the non‐chalk group to Upper Normandy individuals. Hierarchical analyses identified further substructure within the two main groups of rivers. Within the non‐chalk rivers there were multiple, distinct groups of populations. For instance, within the French non‐chalk rivers, three groups where identified—Lower Normandy, Brittany and southern France (Figure 3). Within the British and Irish non‐chalk rivers, the inner Bristol Channel rivers (Twyi, Severn, Wye and Usk) were distinct from other geographically proximate southwest Britain rivers (i.e., Camel, Torridge and Eastern Cleddau). The chalk rivers of the Hampshire Basin and Upper Normandy were distinct groups with little evidence of structuring within each region (Figure 3).

DAPC analysis showed the same strong split as the STRUCTURE analysis (Figure 3, Figure S8). Within each of the two main clusters of individuals, there is separation between the British and French rivers (Figure S6). This analysis also highlighted that two of the adult salmon sampled from Upper Normandy rivers (one each from the Arques and Canche) seem to be strays from the western non‐chalk cluster (Figure S9). The stray into the Arques (ARQ11) appears to have originated from either a Lower Normandy or a Breton river, while the Canche fish (CAN30) is likely a stray from one of the southwest English rivers. The chalk‐only DAPC shows a weak split between the eastern (Test, Itchen and Meon) and western (Frome, Piddle, Avon, Stour) Hampshire Basin rivers (Figure S8) – a distinction that is not apparent in the STRUCTURE results.

### 3.5 Identification of Conservation Units

**FIGURE 4:**
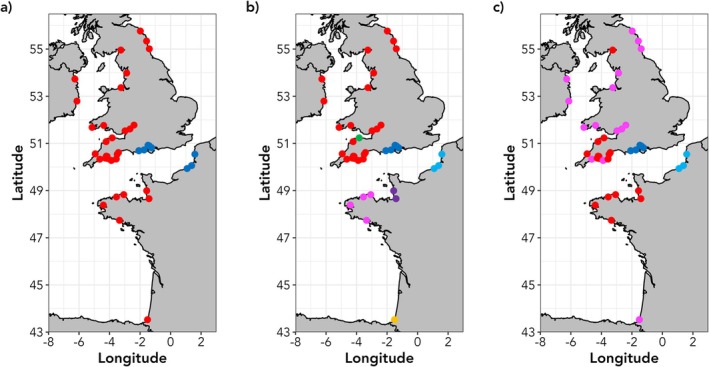
Maps showing designation of conservation units in 42 Atlantic salmon rivers. (a) distribution of Evolutionarily Significant Units, based on all loci datasets (b) Management Units, based on neutral loci datasets. (c) distribution of Adaptive Units based on the outlier loci dataset. Neighbour‐joining dendrograms, on which maps are based are given in Figure S10.

The population‐based NJ dendrograms identified multiple groups of rivers that could be designated as distinct conservation units (Figure 4, Figure S10). Conservatively, based on the data for all 93 loci, we identified two main groupings of rivers that could be classified as ESUs (Figure 4a, Figure S10a). The neutral‐only loci datasets identified seven groupings that could be designated as MUs, namely Upper Normandy, Lower Normandy, Brittany, Hampshire Basin, and western and northern British Isles, with two distinct rivers (ELY and ADR) potentially being single river MUs (Figure 4b, Figure S10b). We distinguish the Brittany and Lower Normandy MUs on the basis of previously published microsatellite data (Perrier et al. 2011). The outlier loci again identified four main groupings. The Hampshire Basin and Upper Normandy rivers were again distinct AUs (Figure 4c, Figure S10c). The rivers of southwest England and Brittany, along with the Dee and Eden, were a third AU, and the remaining British Isles rivers with the Adour constituted the final AU.

### 3.6 Conservation Priorities

**FIGURE 5:**
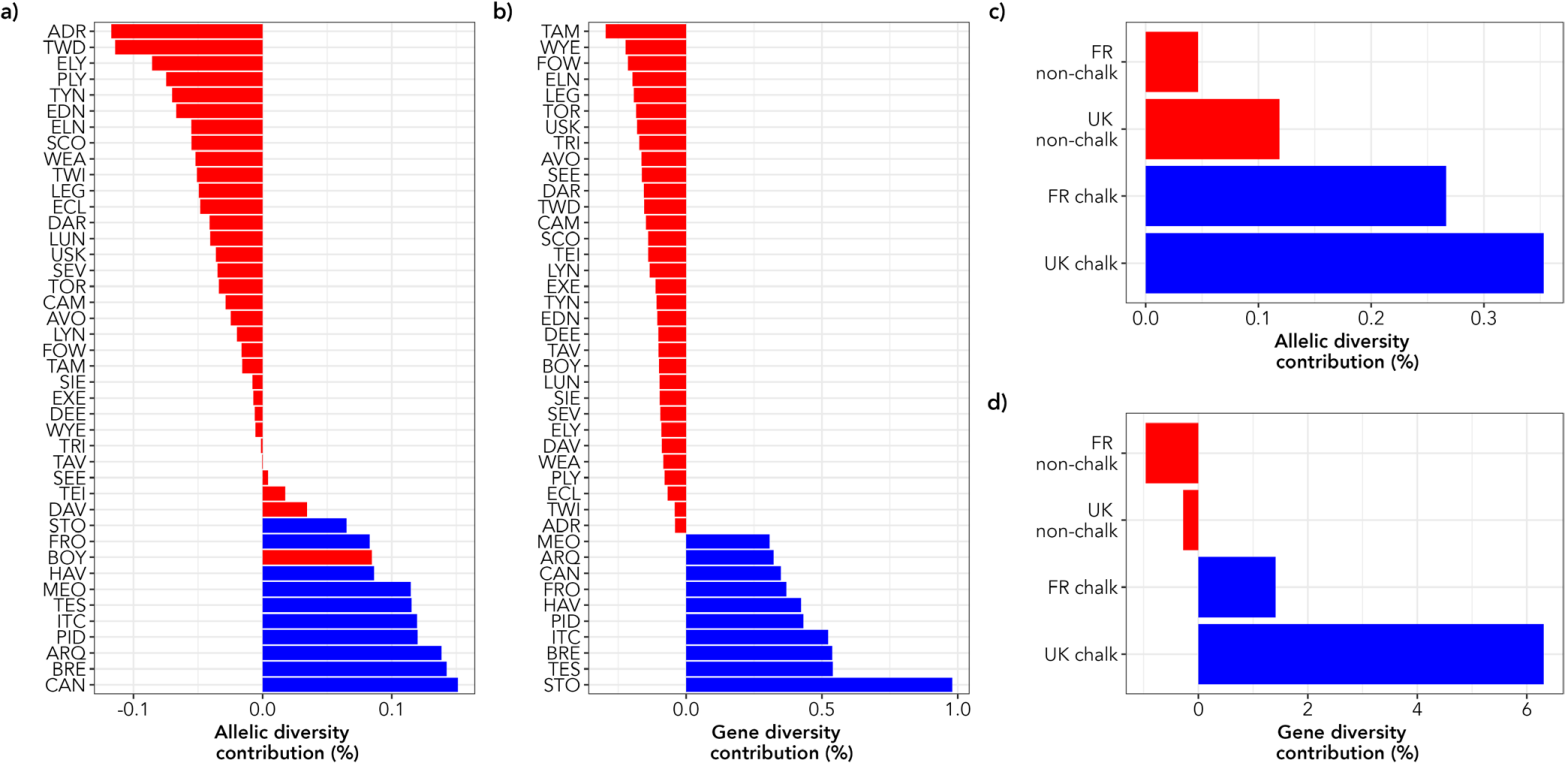
Conservation priorities for 42 Atlantic salmon rivers and four major regional groups of rivers. (a–d) Contribution of 42 rivers (a and b) and four regional groups of rivers (c and d) to total allelic (AT) and gene (HT) diversity. Rivers/regions are sorted from lowest to highest contribution with non‐chalk rivers shown in red and chalk rivers in blue.

Results for the expected contribution to diversity measure showed that only 14 and ten rivers made positive contributions to total allelic (*A*_*T*_) and gene diversity (*H*_*T*_), respectively (Figure 5a,b, Figure S11). For allelic diversity, four non‐chalk and all ten chalk rivers had a positive contribution to the metric. For gene diversity, only the Hampshire Basin and Upper Normandy rivers made positive contributions (Figure 5b). At a regional level, the Hampshire Basin rivers had the largest influence on both metrics, especially for total gene diversity, where the contribution was in excess of 6% (Figure 5c,d). The Hampshire Basin populations also had a negative contribution to both within‐region metrics (*H*_*S*_ and *A*_*S*_; Figure S11), suggesting all seven populations have low within‐river diversity. The Hampshire Basin and Upper Normandy rivers also made positive contributions to *D*_*G*_ and *D*_*A*_ (Figure S11), signifying that they contribute substantially to both between‐river and between‐regional diversity.

## 4 Discussion

Here we present results on the analysis of samples from 42 Atlantic salmon rivers from the British Isles and France with a low‐density SNP panel. For the first time, samples from chalk rivers in southern England and northern France are incorporated into the same study. Multiple analyses of the genetic data confirmed the strong differentiation of these English Channel chalk river salmon from those from non‐chalk rivers, with outlier tests of SNP data suggesting that this could be due to local adaptation to water chemistry. We identified two potential Evolutionarily Significant Units, with the chalk river salmon also being identified as conservation priorities based on their unique and substantial contributions to allelic and gene diversity.

### 4.1 Strong Regional Genetic Structuring

The results suggest that there is strong hierarchical structuring of the genetic diversity present within the salmon rivers investigated here, with four main regional genetic groups: English chalk, French chalk, English non‐chalk, French non‐chalk populations, with further subdivision being present within both English and French non‐chalk river populations as observed in many previous studies of Atlantic salmon. Indeed, such hierarchical structuring is a common pattern in Atlantic salmon in which, at the broadest scales, genetic groups can often span hundreds of kilometres of coastline (Bradbury et al. 2014; Cauwelier, Verspoor, et al. 2018; Finnegan et al. 2013; Gilbey et al. 2018; Griffiths et al. 2010; Wennevik et al. 2019). Differentiation between rivers within each of the four main groups in the current study was generally low; the average pairwise *F*_ST_ between rivers within each of the four groups was all less than 0.035, indicative of low differentiation (Hartl and Clark 1997).

Anadromous salmonids often form metapopulations, where rivers are connected by varying levels of migration between them (Lamarins et al. 2024; Schtickzelle and Quinn 2007). For instance, using otolith chemistry, Fontaine et al. (2025) found frequent dispersal of salmon between three southern French salmon rivers separated by 300 km of coastline, while salmon straying from the River Imsa (Norway) predominately entered rivers within 60 km of the river mouth (Jonsson et al. 2003). Patterns suggestive of frequent dispersal of fish between rivers are also apparent in our results. Salmon from several geographically proximate rivers showed extremely low levels of differentiation (< 0.007; i.e., the Test and Itchen and Tamar and Lynher), indicative of high numbers of straying fish between adjacent rivers. Additionally, there were many cases of pairs of populations within genetic groups demonstrating low differentiation despite being separated by several hundreds of kilometres. For example, the coastal geographic distance between the rivers Exe and the Dee is approximately 700 km, while pairwise *F*_ST_ between the salmon populations in these two rivers was 0.02.

### 4.2 Strong Divergence Between Chalk and Non‐Chalk Rivers

In agreement with previous studies utilising microsatellite analysis (Finnegan et al. 2013; Griffiths et al. 2010; Ikediashi et al. 2018; Perrier et al. 2011), SNP markers reaffirmed the uniqueness of the chalk stream salmon populations, with multiple analyses highlighting the divergence between fish residing in rivers flowing into the western and eastern parts of the English Channel. For instance, the salmon in southwest England and Brittany in northwest France are more closely related to each other than they are to their nearest neighbours further east along each respective coastline. Interestingly, these patterns in the genetic data correspond precisely with the underlying geology/water chemistry experienced by each population. Brittany and southern Devon/Cornwall are dominated by Devonian age bedrock with granitic intrusions, resulting in more acidic river water (pH ≤ 7) with low conductivity. Additionally, the upland areas of Brittany, Devon and Cornwall are dominated by blanket peat bog, reinforcing the acidic nature of river water in these areas. Further east along both coasts, in Normandy and southern England, the underlying geology is dominated by Cretaceous era limestones and chalks, resulting in river water with higher pH above 7.

Multiple, interacting factors help determine the chemical composition of river water. Of particular importance is underlying geology which has a strong influence on pH, conductivity and concentrations of dissolved ions (Jarvie et al. 2002; Liu et al. 2000; Rothwell et al. 2010). It has been suggested that the geological characteristics, and therefore chemical characteristics, of river catchments may be an important factor in determining the accuracy of homing through olfactory‐based imprinting during smolting (Keefer and Caudill 2014) and may help to maintain regional population structure via reduced straying of fish between regional groups of rivers (Almodóvar et al. 2023; Bourret et al. 2013).

Additionally, underlying geology and its effect on river water chemistry has been proposed to be a selective agent in the process of local adaptation in Canadian Atlantic salmon and English populations of brown trout ( *Salmo trutta* ) exposed to high levels of heavy metals (Bourret et al. 2013; Paris et al. 2024). It is interesting to note that the hierarchical genetic structure found here in Atlantic salmon in Channel rivers also occurs in brown trout populations inhabiting rivers flowing into the Channel, with these patterns also having been linked to underlying geology (King et al. 2024). The locations of transitions between groups being coincident in both species reinforces the suggestion that underlying geology and/or water chemistry could be playing an important role in driving local adaptation in salmonids residing in rivers entering the English Channel.

Finally, the finding of multiple SNPs under divergent selection and associated with environmental variables adds strength to the assertion that the observed patterns may be driven by local adaptation. The majority of outlier SNPs showed strong allele frequency differences between chalk and non‐chalk rivers with some loci for example, Ssa_58789 and Ssa_24091, being almost completely monomorphic in the non‐chalk populations but polymorphic in chalk river fish. Unfortunately, the strongest candidate SNP (Ssa_69865) is found in a non‐coding region on linkage group (LG) ssa13. The closest gene, integrator complex subunit 2 (ints2), is approximately 48,000 base pairs upstream of the Ssa_69865 polymorphism.

We discovered that a SNP linked to the gene SIX homeobox 6 (six6) was under divergent section in the full dataset and non‐chalk‐only outlier tests, with the region containing this polymorphism having been shown to be under selection across both the North American and European ranges of Atlantic salmon (Barson et al. 2015; Cauwelier, Gilbey, et al. 2018; Kess et al. 2024; Pritchard et al. 2018; Zueva et al. 2021). This region, on LG ssa09, has been implicated in variation in adult run timing in Scottish salmon populations (Cauwelier, Gilbey, et al. 2018) and age at maturity in Scandinavian salmon (Barson et al. 2015) and suggests that there may be differences in these two life history metrics across the range of the populations sampled here. For instance, the rivers of northeast England tend to have a higher proportion of multi‐sea winter (MSW) salmon in their rod catches than the rivers of southern and southwest England (Environment Agency 2024). Likewise, the rivers of southern France, including the Adour, are dominated by MSW salmon compared to the rivers of Normandy and Brittany (Bal et al. 2017; Le Cam et al. 2015). The highest MAF for Ssa_six6 were found in the rivers of the Bristol Channel, a major inlet separating south Wales from southwest England. The rivers of the inner Bristol Channel (Usk, Wye and Severn) are atypical from the majority of the rivers studied here, being large catchments where adult salmon must undertake extensive freshwater migrations (> 100–300 km) to reach spawning grounds. Indeed, the rod catch of these rivers are dominated by large multi‐sea winter salmon (Environment Agency 2024). Further highlighting the significance of this genomic region, six6 has also been found to be an important determinant of age at maturity in Pacific salmonids (Waters et al. 2021; Willis et al. 2020). With the exception of six6, based on the small number of loci screened here we cannot yet identify any obvious relationship between the outlier loci and any biological function that could account for the distinction between chalk and non‐chalk salmon. However, the outlier loci are spread across multiple chromosomes and possibly indicate that the differences between chalk and non‐chalk salmon are polygenic in nature.

Given the strong observed population structure, substantial locus‐specific variation in *F*_ST_ might be expected under neutrality due to drift alone. Consequently, we recognise that elevated *F*_ST_ at individual loci could reflect the demographic history of the populations studied here rather than divergent selection. We therefore interpret the loci identified as outliers and environment‐associated loci identified as candidates for selection and suggest their role should be evaluated in the context of the strong genome‐wide differentiation observed between chalk and non‐chalk populations. Additional investigation, using reduced representation or whole genome sequencing, will be required to explore both the molecular mechanisms underlying local adaptation and the demographic history of chalk salmon.

### 4.3 Low Divergence Within Genetic Clusters

Anadromous species, such as salmon and trout, are known for their ability to home to their natal river after spending time feeding at sea. This spawning philopatry has profound evolutionary consequences, often leading to reduced gene flow between populations and giving rise to the within‐river genetic structuring often observed in salmonid species (Dillane et al. 2008; Pritchard et al. 2018). However, straying of fish to non‐natal rivers does also occur (Griffiths et al. 2011; Perrier et al. 2011) and is recognised as an important evolutionary feature of salmonids, buffering populations against spatial and temporal variation in local environments (Keefer and Caudill 2014).

Multiple analyses suggest relatively low levels of divergence between salmon populations within each of the four main groups of rivers. Pairwise *F*_ST_ values indicate very low levels of differentiation, especially between geographically proximate rivers such as the Frome and Piddle (*F*_ST_ = 0.006) and the Seé and Sienne (*F*_ST_ = −0.003). Moreover, while DAPC and STRUCTURE analyses suggest that there may be some genetic structuring within the Hampshire Basin chalk rivers, there was only a limited geographical component to this diversity (a relatively weak split between salmon populations in the eastern and western Hampshire Basin rivers).

The freshwater homing of salmon is driven primarily by olfactory imprinting of natal waters, with parr/smolts ‘learning’ the chemical cues of their natal stream prior to seaward migration (Hasler et al. 1978). Increased straying between rivers with similar water chemistry profiles has been found for Canadian Atlantic salmon populations (Bradbury et al. 2014). Given that all the Hampshire Basin rivers originate from the same chalk aquifer system (Allen 2017), the low divergence between salmon populations in these rivers may reflect a high degree of chemical similarity of the water in each of the Hampshire Basin rivers (at least with regard to the homing cues recognised by Atlantic salmon).

### 4.4 Genetic Signal of Historic Stocking in Upper Normandy Populations

The DAPC and STRUCTURE analyses showed clearly that two individuals sampled from two French chalk rivers assigned genetically to non‐chalk rivers. These individuals were sampled as adults and two plausible explanations can be advanced to explain this observation. The first is that the two fish in question are strays from non‐chalk rivers. Straying is a common phenomenon in anadromous salmonids, facilitating colonisation of new habitats and gene flow between rivers (Lamarins et al. 2024), with well‐documented examples of both short‐ and long‐distance movements of salmon between rivers within our study area having been reported previously (Griffiths et al. 2011; Leunda et al. 2013; Perrier et al. 2010). Alternatively, these fish might constitute the remnants of historical stocking programmes. Upper Normandy rivers experienced ‘high’ stocking intensity up to 1990, predominately with salmon from rivers on the east coast of Scotland (Perrier et al. 2013). Previous microsatellite‐based analysis suggested that the genetic effects of this stocking were short‐lived in these rivers (Perrier et al. 2013). In contrast, however, our SNP data suggest a longer‐lasting impact of stocking on genetic diversity within Upper Normandy salmon, with the rivers of this region having the highest values for basic measures of diversity (H_O_ & H_S_) calculated in the current study and the STRUCTURE analysis showing a significant contribution of non‐chalk ancestry.

### 4.5 Salmon Conservation Units

Genetic and genomic data have been used to delimit Conservation Units (ESUs, MUs and AUs) in salmonids that might represent important components of diversity in need of specific conservation and management measures (Lehnert et al. 2023; Waples 1991; Xuereb et al. 2022). A broad definition of an ESU is a group of populations that merit separate management and conservation as a consequence of genetic and ecological distinctiveness (Allendorf et al. 2022). Some definitions also include a degree of geographic isolation (Funk et al. 2012). Our results indicate that the Atlantic salmon rivers studied here could be conservatively classified into two distinct Evolutionarily Significant Units (chalk and non‐chalk) on the basis of genetic, ecological and geographic distinctiveness. Both of these ESUs can be further subdivided into multiple distinct MUs. However, to accurately delimit management units, a more exhaustive sampling of populations in the study area, particularly for northern France, is required. Similarly, given the small number of outlier loci identified here, a more extensive genomic analysis will be needed to accurately define the adaptive units across the regions investigated here.

### 4.6 Chalk Salmon as a Distinct Sub‐Species?

The criteria that are used to distinguish ESUs are similar to those used to delineate a subspecies. Taylor et al. (2017) defined a subspecies as ‘a population, or collection of populations, that appears to be a separately evolving lineage with discontinuities resulting from geography, ecological specialization, or other forces that restrict gene flow to the point that the population or collection of populations is diagnosably distinct’.

The chalk stream salmon appear to fulfil all three requirements. They constitute a genetically distinct and definable unit found in a geographically restricted area. The rivers they inhabit have well‐defined consistent geochemical, morphological and hydrodynamic characteristics (namely all the criteria which define a chalk stream); the lack of evidence of natural straying of salmon with allelic profiles characteristic of non‐chalk rivers into chalk stream populations suggests that these geochemical and hydrodynamic characteristics may be acting to reduce the fitness of any incoming strays and, ergo, resident populations are likely strongly locally adapted to their particular environment. They (the chalk stream salmon—or, more precisely, fish with their genotypes and allelic profiles) are not found anywhere outside the well‐defined geographic region described. Assignment to regions is robust and evidence of straying is virtually absent; in the English chalk stream populations there is no evidence of any successful straying leading to gene flow between chalk and non‐chalk populations, while in French populations, relatively recent non‐triploid stocking may explain the residual presence of some ‘non‐chalk’ alleles in these populations (Perrier et al. 2013).

To date, there appears to have been little appetite to formally recognise subspecies within *Salmo salar* L., and the ESU concept has been largely promoted for the purposes of genetically informed salmon management, especially in North America (Lehnert et al. 2023; Waples 1995). In contrast, while various species and subspecies of European trout have been recognised within the genus *Salmo* (Segherloo et al. 2021), the taxonomy of Atlantic salmon appears to have received considerably less attention.

Currently there has been no formal subspecific classification of any groupings within Atlantic salmon, although some authors have proposed recognition of the eastern and western Atlantic populations as two separate subspecies, *Salmo salar salar* and *S. salar sebago*, respectively (King et al. 2007). These fish exhibit substantial divergence for both mitochondrial (King et al. 2007) and nuclear DNA (Lehnert et al. 2020), including distinct chromosomal differences (Brenna‐Hansen et al. 2012). Based on ~184,000 genome‐wide SNPs, Lehnert et al. (2020) found that mean *F*_ST_ between eastern and western populations of Atlantic salmon was 0.261 and stated that their results warranted the designation of each continental population as subspecies. The degree of differentiation found by Lehnert et al. (2020) is almost identical to that found between the chalk and non‐chalk salmon populations studied here (mean pairwise *F*_ST_ = 0.254). Of course, the limitations inherent in the use of SNP panel comprising 93 genomic loci, as used in the current study, can only ever give a limited picture of levels of divergence, both across the genome and between populations (compared to whole genome analyses), more extensive genomic studies are needed to fully address the intraspecific taxonomy of salmon in this region.

Taking the genetic, ecological and geographical data into consideration, we suggest that the Atlantic salmon populations of the chalk streams meet the criteria for consideration as a subspecies within the species *Salmo salar* L. and suggest the taxonomic name *Salmo salar calcariensis* for this ESU. Certainly, the importance of recognising a genetically definable sub‐specific lineage within Atlantic salmon, *Salmo salar* L., is in keeping with other recent studies on salmonids, for example, *Salmo trutta* L., in Europe (Aparicio et al. 2025; Kaba et al. 2025). In these studies, the formal recognition of distinct sub‐specific taxa using genome‐wide SNP and microsatellite analyses has proven critical in aiding the management and conservation of threatened genetically distinct trout ESUs, which might otherwise have been grouped with non‐at‐risk, cosmopolitan taxonomic lineages, thereby depriving them of the critical recognition needed for active protection.

Finally, it is important to recognise differences in the ways in which genetic data are currently used in conservation and management legislation in North America and the UK. Unlike wildlife protection legislation in the USA (Waples 1995), Canada (Lehnert et al. 2023) and other regions (Hoelzel 2023), ESUs are not currently recognised in the UK Habitats Directive, the main legislation responsible for conservation of species and habitats in the United Kingdon (J. Gray, Wildfish Trust, personal communication). However, subspecies can be specified in the Habitats Directive. In the case of chalk stream salmon, we anticipate that their formal designation as a distinct subspecies will in fact benefit their conservation more than being designated as an ESU.

### 4.7 The Conservation Value of Chalk Stream Salmon

It is clear that concerted conservation action is required to halt the continuing decline in both the numbers and genetic diversity observed across the species' range of Atlantic salmon (Lehnert et al. 2019). Moreover, as different environmental stressors act across different parts of the species' range, the continuing preservation of healthy regional populations is a key concern for conservation management of the species (von Takach et al. 2024).

In particular, the UK chalk stream salmon populations are highlighted for their unique genetic make‐up and the importance of their contribution to the overall diversity of the species. All had negative contributions to both H_S_ and A_S_ (Figure S8), suggesting the populations in the seven studied rivers have low within‐river diversity. This is reinforced by these populations generally having the lowest observed and expected heterozygosities and locus polymorphism of the populations studied. The significant positive contributions to between‐population metrics and negative within‐population contributions suggest that these rivers have been affected by long‐term isolation, genetic drift and/or bottlenecks (von Takach et al. 2024). Given that the populations in the Hampshire Basin rivers are in continued decline (Environment Agency 2024) and that some rivers have experienced significantly poor recruitment in recent years (UK Environment Agency & GWCT, personal communications), there is a need for enhanced conservation and protection above that already afforded under United Kingdom law.

### 4.8 Conclusions

In summary, we demonstrate that the chalk stream salmon of southern England and northern France constitute a unique component of the Atlantic salmon meta population; they contain a distinctive and substantial component of the genetic diversity of the species and this degree of genetic novelty is such that they constitute a distinct ESU, linked primarily to the underlying geology and water chemistry of their local habitat. As such, their extirpation would result in the loss of > 6% of the overall genetic diversity of the species (based on the populations included in the current study) and we reiterate the urgent need to preserve them and the rivers in which they reside.

Genetically distinct populations should be prioritised for conservation as these often represent unique evolutionary lineages, within the overall metapopulation structure of species such as Atlantic salmon. Indeed, one of the overarching strengths of conservation genetics is the ability to define evolutionarily divergent units (populations) within a species that warrant recognition and separate/bespoke management. Chalk salmon appear to represent such a unit and may be potentially viewed either as a distinct ESU or, potentially, as a new subspecies.

## Funding

This research was funded by the European Union Interreg France (Channel) England programme project ‘Salmonid Management Around the Channel’ (SAMARCH) with additional funding from Southern Water, UK.

## Conflicts of Interest

The authors declare no conflicts of interest.

## Supporting information


**Table S1:** Basic measures of genetic diversity for Atlantic salmon from 42 UK, Irish and French rivers screened for variation at 93 single nucleotide polymorphism (SNP) markers. Risk categories are taken from the NASCO Rivers Database (https://nasco.int/rivers‐database/).
**Table S2:** Details of genome location for each of the 94 single nucleotide polymorphism loci used in this study. The first 15 loci were found to be under selection in *F*_ST_‐based outlier tests (Bayescan, fdist and hierarchical fdist) and/or associated with environmental variables in Redundancy Analyses (RDA) and BayeScEnv.
**Table S3:** Details of SNP loci removed from the non‐chalk and chalk hierarchical datasets. River codes are as given in Table S1.
**Figure S1:** Results for selection tests for chalk‐only and non‐chalk‐only datasets. (a, b) Bayescan results. The dashed blue line represents the log_10_ PO value above which selection on loci is considered significant. (c, d) fdist results. Dashed lines are the upper and lower 99% confidence level of the simulated neutral distribution. Loci under either divergent or balancing selection are shown in red with loci under divergent selection labelled with locus name. (a) & (c) results for the non‐chalk river data set of 89 SNPs screened in 32 rivers; (b) & (d) results for the chalk river data set of 88 SNPs screened in 10 rivers.
**Figure S2:** Heatmap showing the minor allele frequencies for 15 SNPs found to be under divergent selection in outlier tests and associated with environment in Redundancy Analyses (RDA). Numbers above each column denotes in which test each locus was identified (1—Bayescan, 2—fdist, 3—hierarchical fdist, 4—unconditioned RDA, 5—RDA conditioned by geographical distance from northern‐most river, 6—BayeScEnv).
**Figure S3:** Results of hierarchical fdist *F*_ST_ model based on two and four groupings of rivers. (a) chalk v non‐chalk and (b) UK chalk v FR chalk v UK non‐chalk v FR non‐chalk. Dashed lines are the upper and lower 99% confidence level of the simulated neutral distribution.
**Figure S4:** Results of Redundancy Analysis conditioned on geographic distance from northern‐most river. (a) Plot of conditioned redundancy analysis axes 1 v 2. Black vectors represent loadings for the seven retained bioclim variables. River codes are as given in Table S1. (b–d) Plot of minor allele frequency for loci Ssa_67740 & Ssa_30724 versus standardised bio17 (precipitation of the driest quarter) values and locus Ssa_87179 versus standardised bio10 (mean temperature of the warmest quarter) values. The red line represents the linear regression, and the grey shaded area is the 95% confidence interval.
**Figure S5:** Results of BayeScEnv. Plots are the −log10(q‐value) for each of 93 SNP loci for each of 19 bioclim environmental variables. The dotted line represents a q‐value of 0.05 (−log10 = 1.3) at which associations are considered significant.
**Figure S6:** Heatmap showing pairwise Weir & Cockerman's *F*_ST_ between 42 British, Irish and French Atlantic salmon rivers. River codes are as given in Table S1.
FigureS7:
Results for Evanno et al. (2005) delta *K* (Δ*K*) for the hierarchical STRUCTURE analyses of genetic structuring in 42 Atlantic salmon rivers.
**Figure S8:** Discriminant Analysis of Principal Components (DAPC) analyses for Atlantic salmon from UK, Irish and French rivers. Each dot represents a sampled individual fish. River codes are as given in Table S1 and dots are coloured as given in Figure 3a. (a) – non‐chalk river data set (1124 individuals from 32 rivers) and (b) – chalk river data set (441 individuals from 10 rivers).
**Figure S9:** Discriminant Analysis of Principal Components (DAPC) analyses for Atlantic salmon from 42 UK, Irish and French rivers highlighting the two suspected strays (CAN30 & ARQ11) from non‐chalk rivers into two French chalk rivers. Each dot represents a sampled individual fish. River codes are as given in Table S1 and dots are coloured as given in Figure 3a.
**Figure S10:** Population‐based Neighbour‐joining dendrograms for (a) all, (b) neutral and (c) outlier datasets. River codes are as given in Table S1 and branches are coloured as given in Figure 4.
**Figure S11:** Contribution of 42 Atlantic salmon rivers and four major regional groups of rivers to genomic diversity. Contributions are partitioned into within‐population/group (*A*_*S*_ and *H*_*S*_), between‐population/group (*D*_*A*_ and *D*_*G*_) and total (*A*_*T*_ and *H*_*T*_) components of allelic and gene diversity, respectively.

**Table S4:** Population pairwise Weir & Cockerman's *F*_ST_ (below diagonal) between 42 UK, Irish and French Atlantic salmon rivers. River codes are as given in Table S1. Significance, based on 999 bootstrap replicates, is given above the diagonal (ns, not significant, *0.05 > *p* < 0.01, **0.01 > *p* < 0.001, ***p > 0.001).

## Data Availability

The data that support the findings of this study are openly available in Dryad at https://datadryad.org/, reference number doi:10.5061/dryad.2bvq83c3n.
